# Supplementary material for: Community Perceptions of a Multilevel Sanitation Behavior Change Intervention in Rural Odisha, India
Source: Int J Environ Res Public Health. 2020 Jun 22;17(12):4472. doi: 10.3390/ijerph17124472 (PMC7345262; doi:10.3390/ijerph17124472)
Supplement: Supplementary file 1 [file ijerph-17-04472-s001.pdf]

International Journal of *Environmental Research and Public Health*

MDPI

Article

Community perceptions of a multi-level sanitation behavior change intervention in rural Odisha, India

Renee De Shay[1], Dawn L. Comeau[1], Gloria D. Sclar[2], Parimita Routray[3], Bethany A. Caruso*[2, 4]

[1] Department of Behavioral, Social, & Health Education Sciences, Rollins School of Public Health, Emory University, Atlanta, GA 30322, USA; reneedeshay@gmail.com; dcomeau@emory.edu

[2] Gangarosa Department of Environmental Health, Rollins School of Public Health, Emory University, Atlanta, GA 30322, USA; gloria.sclar@emory.edu; bethany.caruso@emory.edu

[3] Independent Consultant, Bhubaneswar, Odisha, India; rparimita@gmail.com

[4] Hubert Department of Global Health; Rollins School of Public Health, Emory University, Atlanta, GA 30322, USA

* Correspondence: bethany.caruso@emory.edu

Received: date; Accepted: date; Published: date

Abstract: While latrine coverage is increasing in India, not all household members use their latrines. Cost-effective, culturally appropriate, and theory-informed behavior change interventions are necessary to encourage sustained latrine use by all household members. We qualitatively examined community perceptions of sanitation interventions broadly, along with specific impressions and spillover of community-level activities of the Sundara Grama latrine use behavior change intervention in rural Odisha, India. We conducted sixteen sex-segregated focus group discussions (n=152) in three intervention and three non-intervention villages and thematically analyzed the data. We found Sundara Grama was well-received by community members and considered educative, but perceptions of impact on latrine use were mixed and varied by activity. Intervention recruitment challenges prevented some, such as women and households belonging to lower castes, from attending activities. Spillover occurred in one of two non-intervention villages, potentially due to positive relations within and between the non-intervention village and nearby intervention village. Community-level sanitation initiatives can be hindered by community divisions, prioritization of household sanitation over community cleanliness, and perceptions of latrine use as a household and individual issue, rather than common good. Community-centered sanitation interventions should assess underlying social divisions, norms, and perceptions of collective efficacy to adapt intervention delivery and activities.

Keywords: WASH, qualitative research, latrine use, process evaluation, gender, collective efficacy

Table S1. Descriptions of Sundara Grama Behavior Change Activities

Activity	Level of Change	Aim and Description
		Assessed in this study
Palla	Community	To educate and engage community members on the health and non-health benefits to latrine use through a traditional folk theater performance
Transect walk	Community	To encourage community members to re-evaluate village cleanliness by marking feces with colored powder during an early morning walk through village and defecation fields.
Community meeting	Community	To facilitate community motivation, action, and commitment toward achieving sanitation through an interactive meeting with creation of a community action plan.
		Other activities
Mother's meeting	Group	To provide mothers with practical knowledge on the materials and practices used to safely dispose of infant and young children's feces into latrine.
Household visits	Household	To reiterate and reflect on intervention messages and facilitate household commitment toward achieving sanitation, with distribution of a reminder poster.
Community wall painting*	Community	To publicly display the community-identified goals and action steps for sanitation alongside a painted community map in order to track progress towards the community goals.
Latrine repairs*	Household	To make minor repairs to household latrines so they are functional, safe and usable.

*Not completed at the time of this study.

Table S2. Process Evaluation Elements in FGD Guides as Informed by Sanders et al (2005) [43]

Element	Definition from Saunders[1]	Example FGD Questions
	Intervention and Non-intervention Village FGD Guides	
Satisfaction	"Participant (primary and secondary audiences) satisfaction with program, interactions with staff and/or investigators."	What did you think of [activity name]? How did it make you feel?
Reach	"Proportion of the intended priority audience that participates in the intervention; often measured by attendance; includes documentation of barriers to participation"	Who attended [the activity]? Probe for age, caste, and gender.<br><br>If you were unable to attend or left early, why?
Recruitment	"Procedures used to approach and attract participants at individual or organizational levels; includes maintenance of participant involvement in intervention and measurement components of study."	How did you find out about [activity name]?
	Non-intervention Village FGD Guide	
Context	"Aspects of the environment that may influence intervention implementation or study outcomes; includes contamination or the extent to which the control group was exposed to the program."	Have there ever been sanitation promotion events in this village? Do you think these events were good, why or why not?<br><br>Have you heard about the recent sanitation events in [paired intervention village]?

1. (p. 139)

© 2020 by the authors. Submitted for possible open access publication under the terms and conditions of the Creative Commons Attribution (CC BY) license (http://creativecommons.org/licenses/by/4.0/).
